# Development and evaluation clinical-radiomics analysis based on T1-weighted imaging for diagnosing neonatal acute bilirubin encephalopathy

**DOI:** 10.3389/fneur.2023.956975

**Published:** 2023-02-14

**Authors:** Jinhong Yu, Yangyingqiu Liu, Yuhan Jiang, Bingbing Gao, Jingshi Wang, Yan Guo, Lizhi Xie, Yanwei Miao

**Affiliations:** ^1^Department of Radiology, First Affiliated Hospital of Dalian Medical University, Dalian, Liaoning, China; ^2^Department of Radiology, Dalian Woman and Children's Medical Center (Group), Dalian, Liaoning, China; ^3^GE Healthcare, Life Science China, Shenyang, Liaoning, China; ^4^MRI Research, GE Healthcare, Beijing, China

**Keywords:** radiomics, acute bilirubin encephalopathy, magnetic resonance imaging, nomogram, hyperbilirubinemia

## Abstract

**Purpose:**

To investigate the value of clinical-radiomics analysis based on T1-weighted imaging (T1WI) for predicting acute bilirubin encephalopathy (ABE) in neonates.

**Methods:**

In this retrospective study, sixty-one neonates with clinically confirmed ABE and 50 healthy control neonates were recruited between October 2014 and March 2019. Two radiologists' visual diagnoses for all subjects were independently based on T1WI. Eleven clinical and 216 radiomics features were obtained and analyzed. Seventy percent of samples were randomly selected as the training group and were used to establish a clinical-radiomics model to predict ABE; the remaining samples were used to validate the performance of the models. The discrimination performance was assessed by receiver operating characteristic (ROC) curve analysis.

**Results:**

Seventy-eight neonates were selected for training (median age, 9 days; interquartile range, 7–20 days; 49 males) and 33 neonates for validation (median age, 10 days; interquartile range, 6–13 days; 24 males). Two clinical features and ten radiomics features were finally selected to construct the clinical-radiomics model. In the training group, the area under the ROC curve (AUC) was 0.90 (sensitivity: 0.814; specificity: 0.914); in the validation group, the AUC was 0.93 (sensitivity: 0.944; specificity: 0.800). The AUCs of two radiologists' and the radiologists' final visual diagnosis results based on T1WI were 0.57, 0.63, and 0.66, respectively. The discriminative performance of the clinical-radiomics model in the training and validation groups was increased compared to the radiologists' visual diagnosis (*P* < 0.001).

**Conclusions:**

A combined clinical-radiomics model based on T1WI has the potential to predict ABE. The application of the nomogram could potentially provide a visualized and precise clinical support tool.

## Introduction

Neonatal hyperbilirubinemia, characterized by jaundice, is a common and benign disease in neonates, but is the main cause of hospitalization in the first week after birth (1); neonatal jaundice is the seventh and the ninth cause of neonatal death in the early neonatal period (0–6 d) and late neonatal period (7–27 d), respectively (2, 3). Even though most hyperbilirubinemia patients have a favorable prognosis, some severe cases may cause neurotoxicity and lead to neonatal acute bilirubin encephalopathy (ABE) (4) with a risk of neonatal mortality and life-long neurodevelopmental handicaps (5, 6).

Hyperintensity of the bilateral globus pallidus (GP) on T1-weighted imaging (T1WI) is considered to be a characteristic imaging manifestation of ABE (7, 8). However, the presence of myelin in these structures has also revealed hyperintensity on T1WI, which is easily confused with the changes caused by brain damage in ABE (9). Furthermore, hyperintensity on T1WI of GPs in neonates with ABE may be transient and subtle (7); the lack of objectivity and accuracy necessitates the need to determine GP signals by a radiologist's naked eye only, which may affect the early diagnosis and treatment of ABE, especially for those neonates who may have had early brain damage while the signal of GPs on T1WI has not increased. Therefore, conventional T1WI is insufficient to meet diagnostic needs. Radiomics is an emerging field that translates medical images into quantitative data (10, 11). The internal texture information of the GPs can be mined deeply by extracting high-throughput features, and the biological information can be displayed objectively and quantitatively.

The purpose of this study was to assess the value of a clinical-radiomics model based on T1WI for predicting ABE in neonates and to compare its diagnostic value with experienced radiologists' visual diagnosis results, providing a new tool for the early diagnosis and individualized monitoring of ABE.

## Materials and methods

### Patients

**Figure 1:**
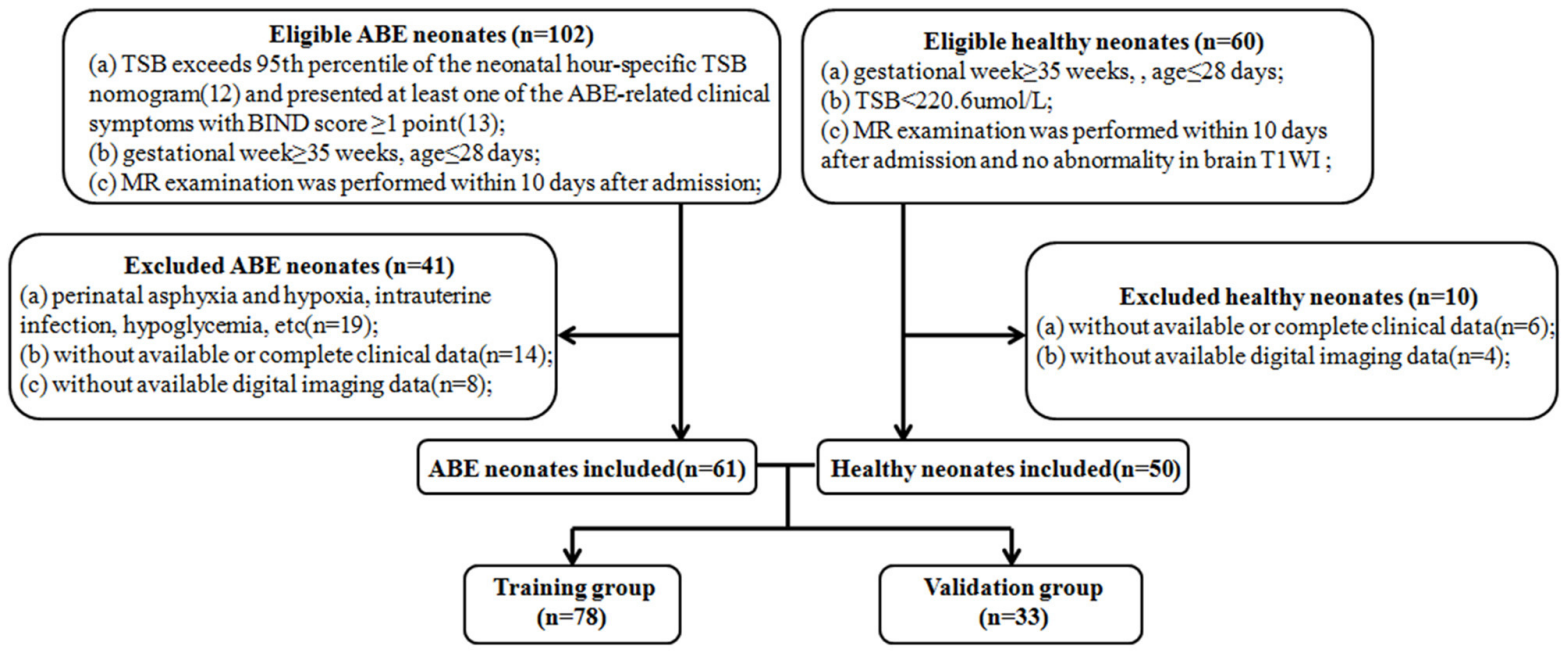
Flow diagrams show the pathway of ABE and healthy neonate inclusion and exclusion. ABE, acute bilirubin encephalopathy; TSB, total serum bilirubin; BIND, bilirubin-induced neurologic dysfunction.

This retrospective study was approved by the hospital ethics committee and the requirement for informed consent was waived because of the retrospective nature of the study. One hundred and eleven neonates were recruited between October 2014 and March 2019. Figure 1 shows the pathway of ABE and healthy neonate inclusion and exclusion. Clinical features including age, gender, weight, gestational week, pregnancy history of maternal, meconium-stained amniotic fluid, premature rupture of membranes, singleton or multiple-birth pregnancy, and type of delivery were collected from all subjects. In order to construct a stable and generalization model, all subjects (*n* = 111) were randomly divided into a training group (*n* = 78) and a validation group (*n* = 33) according to the ratio of 7:3. The training group was used to construct models that were verified by the validation group.

### MRI acquisition

All subjects underwent MRI examination on a GE Signa HDxT 1.5T MRI scanner with a dedicated eight-channel head and neck unite coil. Ten percent chloral hydrate (0.5 mL/kg) was administered *via* anal enema for sedation 30 min before examination. MRI scanning was performed during the deep sedation. The imaging protocol included axial T1WI (TR = 2950 ms, TE = 25 ms, slice thickness = 4 mm, matrix = 288 × 192, NEX = 2, FOV = 22.0 × 22.0 cm).

### Radiologists' visual diagnosis

Radiologist 1 (JY) and radiologist 2 (JW), with 15 and 20 years of experience in neonatal radiology, respectively, independently provided what they thought was the most likely diagnosis for all subjects (*n* = 111) based on T1WI. For the subjects with controversial diagnoses from radiologists 1 and 2, a review was conducted by radiologist 3 with 25 years of experience (YM) to determine the radiologists' final visual diagnosis. Radiologists were blinded to all clinical and diagnosis information except age at the time of MRI scanning. Images were displayed in a random sequence.

### Segmentation and feature extraction

MRI data were transferred to a personal computer and processed based on 3D slicer software (http://www.slicer.org). The regions of interest were manually outlined along the boundary of the bilateral GPs on the slices with the largest area of bilateral GPs and its adjacent upper and lower slices on T1WI. Then, a volume of interest was automatically generated on the software. One hundred and six features of each GP (212 features of bilateral GPs) for each neonate were extracted by 3D slicer software. All radiomics features are summarized in Supplementary material E1.

### Feature selection and prediction model building

#### Clinical feature and model

The clinical features in the training and validation group were compared using an independent samples *t*-test (for normally distributed data) or Mann–Whitney *U*-test (for non-normally distributed data) for continuous variables and a chi-squared test for categorical variables.

Features with *P* < 0.05 in the univariate analysis were selected and fed into the multivariate logistic regression analysis using a backward stepwise elimination method based on Akaike's information criterion. The clinical model was established by applying multivariate logistic regression.

#### Radiomics feature and model

The dimensionality reduction of radiomics features was performed. First, intraclass correlation coefficients were used to assess the intra-observer repeatability of radiomics features. The image segmentation of all cases was performed by observer one with 15 years of experience (JY), and then observer two with 10 years of experience (YL), and used the same method to segment the 30 subjects who were selected from the whole sample based on stratified sampling to reduce possible bias and ensure the reliability of segmentation and feature extraction. Observers were blinded to clinical and group information. Features with intraclass correlation coefficients > 0.90 were retained, which indicated high robustness and satisfactory reproducibility. Second, maximal relevance and minimal redundancy were performed to eliminate the redundant and irrelevant features, and 30 features were retained. Then, the least absolute shrinkage and selection operator algorithm was performed to identify the most valuable features. Finally, the radiomics score (rad-score) was calculated based on the selected radiomics features weighted by their coefficients.

#### Clinical-radiomics model and nomogram

The combined clinical-radiomics model was established to predict the risk of ABE using multivariate logistic regression analysis based on the selected clinical features and radiomics features. To ensure the easy use of the model, the combined clinical-radiomics model was further visualized as a nomogram.

#### Evaluation

The discrimination performances of the radiologists' visual diagnosis, clinical model, radiomics model, and clinical-radiomics model were accessed by using receiver operating characteristic (ROC) curve analysis. The area under the ROC curve (AUC), accuracy, sensitivity, and specificity were calculated. DeLong's test was used to compare the statistical differences between the AUCs of the training and validation groups. The calibration performance of the combined model was tested by using the calibration curves accompanied by the Hosmer–Lemeshow test; *P* > 0.05 were considered good fitness. The clinical benefit for clinical application of the combined model was assessed by using decision curve analysis.

**Figure 2:**
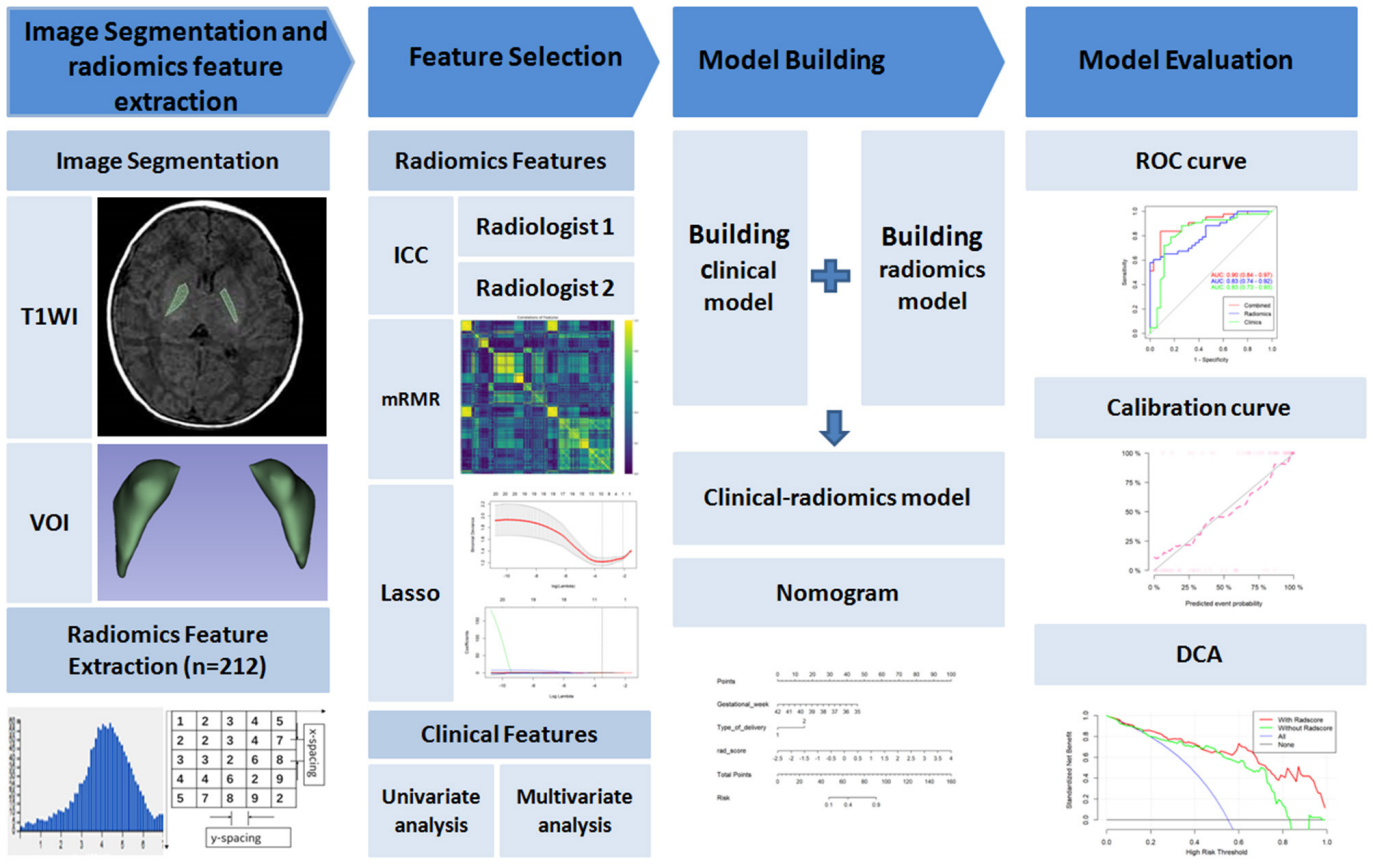
The workflow of clinical-radiomics analysis in the current study. VOI, volume of interest; ICC, intraclass correlation coefficients; mRMR, maximal relevance and minimal redundancy; Lasso, least absolute shrinkage and selection operator; ROC, receiver operating characteristic; DCA, decision curve analysis.

The statistical analyses were performed using R software (v. 3.5.3, http://www.R-project.org), and two-sided *P* < 0.05 were considered to be statistically significant. The workflow is shown in Figure 2.

## Results

### Clinical feature and model

Sixty-one neonates with ABE (median age, 9 days; interquartile range, 7–10 days; 35 male) and 50 healthy neonates (median age, 11.5 days; interquartile range, 6–19.75 days; 38 male) were included for the training and validation groups. Demographic and clinical features data are provided in Table 1.

**Table 1:** Patient clinical features between the training and validation groups.

**Clinical feature**	**Training group (*****n =*** **78)**			**Validation group (*****n =*** **33)**			***P*-value**
	**ABE (*****n** =* **43)**	**HC (*****n** =* **35)**	***P*** **-value**	**ABE (*****n** =* **18)**	**HC (*****n** =* **15)**	***P*** **-value**	
**Age (d)**	8.00 (2.50)	11.00 (11.50)	0.220	9.50 (5.25)	19.00 (4.75)	0.135	0.502
**Gender**			0.168			0.697	0.315
Male	24 (55.81%)	25 (71.43%)		14 (77.78%)	10 (66.67%)		
Female	19 (44.19%)	10 (28.57%)		4 (22.22%)	5 (33.33%)		
**Weight (g)**	3224.42 ± 488.03	3447.00 ± 588.04	0.072	3262.22 ± 603.94	3336.00 ± 645.50	0.737	0.885
**Gestational week (w)**	38.35 ± 1.12	38.56 ± 1.36	0.760	38.42 ± 1.36	38.56 ± 1.51	0.642	0.808
**Pregnancy history of maternal**			0.820			0.482	0.891
Yes	17 (39.53%)	15 (42.86%)		9 (50.00%)	5 (33.33%)		
No	26 (60.47%)	20 (57.14%)		9 (50.00%)	10 (66.67%)		
**Meconium-stained amniotic fluid**			0.078			0.375	0.977
Yes	40 (93.02%)	24 (68.57%)		16 (88.89%)	11 (73.33%)		
No	3 (6.98%)	11 (31.43%)		2 (11.11%)	4 (26.67%)		
**Premature rupture of membranes**			0.649			0.300	0.188
Yes	20 (46.51%)	19 (54.29%)		5 (27.78%)	7 (46.67%)		
No	23 (53.49%)	16 (45.71%)		13 (72.22%)	8 (53.33%)		
**Singleton or multiple-birth pregnancy**			0.198			1.000	0.366
Singleton	43 (100.00%)	33 (94.29%)		17 (94.44%)	14 (93.33%)		
Multiply	0 (0%)	2 (5.71%)		1 (5.56%)	1 (6.67%)		
**Type of delivery**			<0.001^*^			<0.001^*^	0.342
Spontaneous labor	6 (13.95%)	20 (57.14%)		0 (0%)	8 (53.33%)		
Cesarean delivery	37 (86.05%)	15 (42.86%)		18 (100.00%)	7 (46.67%)		
**TSB (umol/L)**	387.84 ± 68.17	-		369.39 ± 40.30	-	0.196	
**BIND score**	3.00 (2.00)	-		3.00 (1.00)	-	0.731	

^*^p < 0.05.

The clinical features were further analyzed by univariate and multivariate logistic regression analysis in the training group (Table 2). In the univariate analysis, gestational week, meconium-stained amniotic fluid, and type of delivery (*P* < 0.05) were included in the multivariate analysis. Multivariate logistic regression analysis indicated that gestational week and type of delivery (*P* < 0.05) were independent risk factors for predicting ABE. The clinical model was established using the above three features.

**Table 2:** Univariate and multivariate logistic regression analysis of clinical features in the training group.

**Clinical features**	**Univariate analysis**		**Multivariate analysis**	
	**Odds ratio**	***P*** **-value**	**Odds ratio**	***P*** **-value**
Gestational week	0.45	<0.001^*^	0.47	0.001^*^
Type of delivery	8.22	<0.001^*^	7.53	<0.001^*^

^*^p < 0.05.

### Radiomics features and model

One hundred features of left GP and 106 features of right GP were considered stable with intra-observer stability (intraclass correlation coefficients ranges: 0.901–1.000 and 0.941–1.000, respectively). These features measured by observer one were selected for subsequent analysis.

There remained ten radiomics features after dimensionality reduction, and the coefficients of these features are presented in Figure E1; these were selected to calculate the corresponding rad-score:


$$\begin{array}{l} \text{Rad}-\text{score }=0.285\;+\;0.805\times \text{Right\_RunEntropy}+0.164\\ \times \text{Left\_Correlation}-0.049\times \text{Right\_SmallAreaLowGrayEmphasis}\\ +0.267\times \text{Right\_SurfaceVolumeRatio}-0.264\times \text{Right\_Imc}1\\ -0.325\times \text{Left\_Maximum}2\text{DDiameterRow}-0.071\\ \times \text{Left\_Skewness}+0.12\times \text{Right\_Correlation}+0.264\\ \times \text{Left\_GrayLevelNonUniformity\_}2+\;0.237\times \text{Left\_Idmn} \end{array}$$

### Clinical-radiomics model and nomogram construction

**Figure 3:**
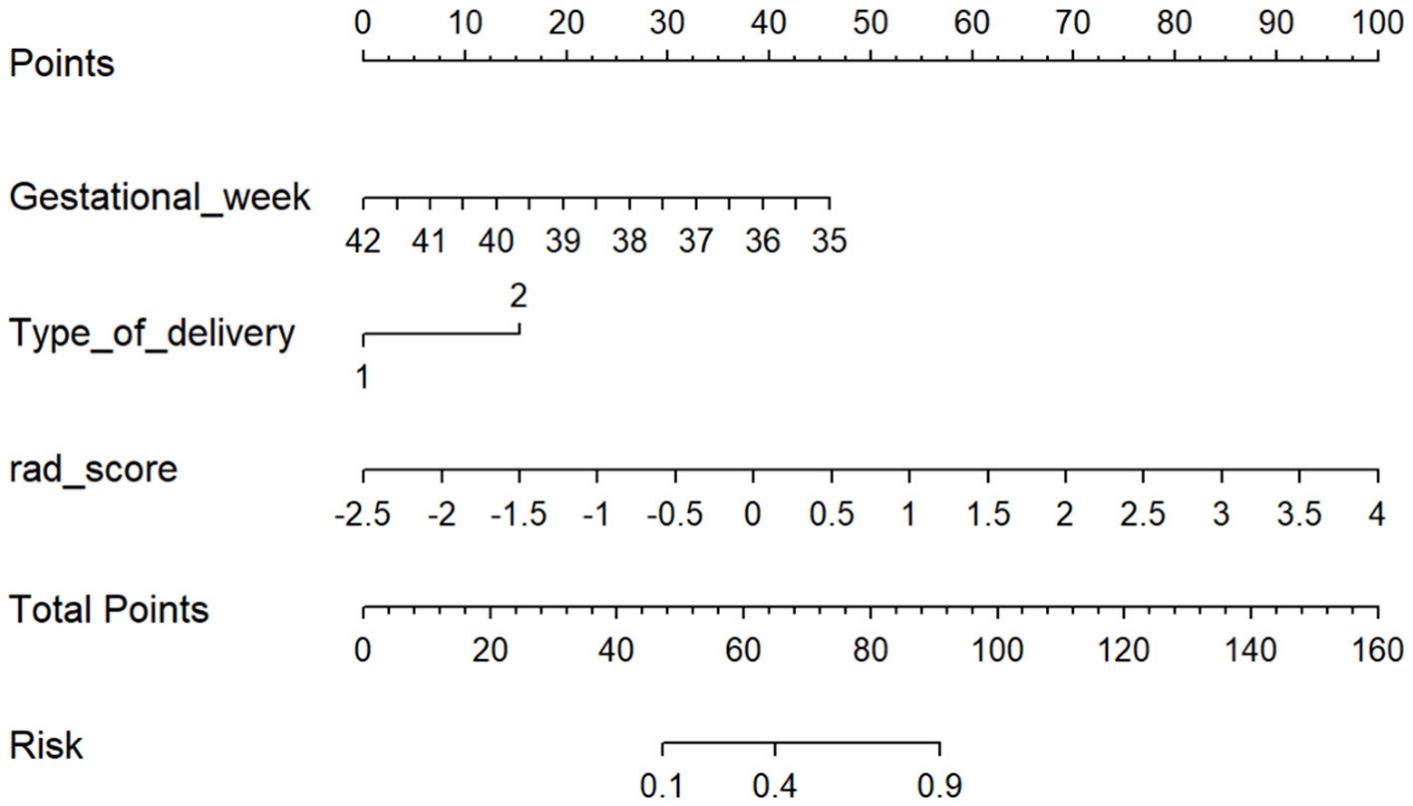
The nomogram based on the clinical-radiomics model. Including gestational week, type of delivery, and rad-score. For each neonate, the total points were calculated by adding up the points of each variable and translated into the risk of ABE. Type of delivery 1 represents spontaneous labor and 2 represents cesarean delivery.

Combined with the clinical features and the rad-score, the clinical-radiomics model was established. Meanwhile, the nomogram was established based on the clinical-radiomics model to individually estimate the risk of ABE for each neonate (Figure 3).

### Model performance evaluation

**Figure 4:**
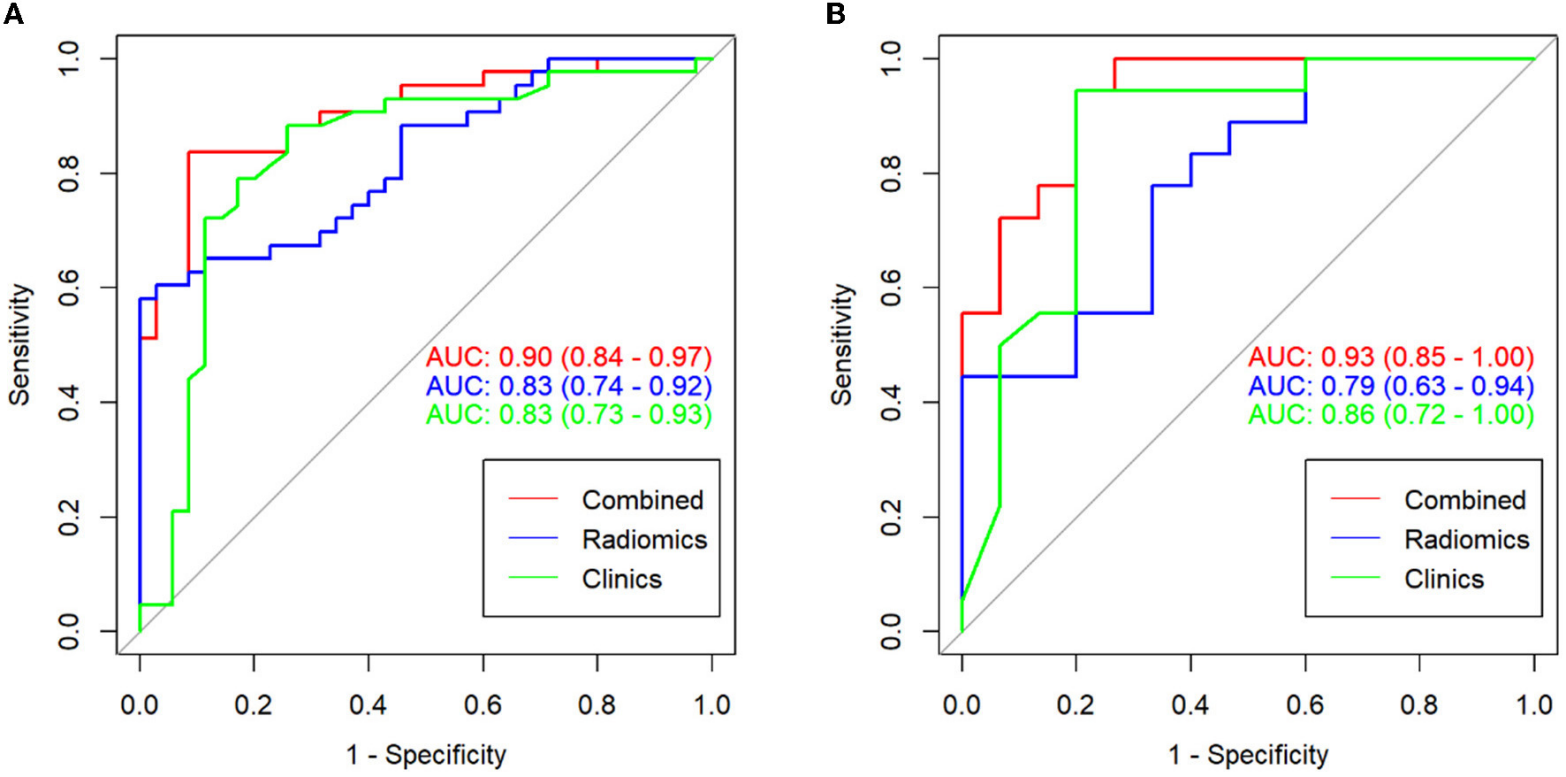
ROC curves for the clinical model, radiomics model, and clinical-radiomics model in the training group **(A)** and the validation group **(B)**.

The ROC curves and discriminative performance of the clinical model, radiomics model, and clinical-radiomics model are shown in Figures 4A, B and Table 3. In the training group, the AUC of the clinical model, radiomics model, and clinical-radiomics model was 0.83 (*P* < 0.001, 95% CI: 0.73–0.93), 0.83 (*P* < 0.001, 95% CI: 0.74–0.92), and 0.90 (*P* < 0.001, 95% CI: 0.84–0.97), respectively, with a sensitivity of 0.884, 0.581, and 0.814, specificity of 0.743, 1.000, and 0.914, positive predictive value of 0.809, 1.000, and 0.921, and negative predictive value of 0.839, 0.660, and 0.800. In the validation group, the AUC of the clinical model, radiomics model, and clinical-radiomics model was 0.86 (*P* < 0.001, 95% CI: 0.72–1.00), 0.79 (*P* = 0.005, 95% CI: 0.63–0.94), and 0.93 (*P* < 0.001, 95% CI: 0.85–1.00), respectively, with a sensitivity of 0.944, 0.778, and 0.944, specificity of 0.800, 0.667, and 0.800, positive predictive value of 0.850, 0.737, and 0.850, and negative predictive value of 0.923, 0.714, and 0.923. DeLong's test showed that there was no significant difference between the training group and the validation group in the clinical model, radiomics model, and clinical-radiomics model (*P* = 0.759, *P* = 0.670, and *P* = 0.542, respectively).

**Table 3:** ROC curves of models in the training and validation group.

**Model**		**Clinical model**	**Radiomics model**	**Clinical-radiomics model**
**Training group**	AUC (95% CI)	0.83 (0.73–0.93)	0.83 (0.74–0.92)	0.90 (0.84–0.97)
	Sensitivity	0.884	0.581	0.814
	Specificity	0.743	1.000	0.914
	Positive predictive value	0.809	1.000	0.921
	Negative predictive value	0.839	0.660	0.800
**Validation group**	AUC (95% CI)	0.86 (0.72–1.00)	0.79 (0.63–0.94)	0.93 (0.85–1.00)
	Sensitivity	0.944	0.778	0.944
	Specificity	0.800	0.667	0.800
	Positive predictive value	0.850	0.737	0.850
	Negative predictive value	0.923	0.714	0.923
***P*** **-value**		0.759	0.670	0.542

ROC, receiver operating characteristic; AUC, area under the curve; CI, confidence interval.

**Figure 5:**
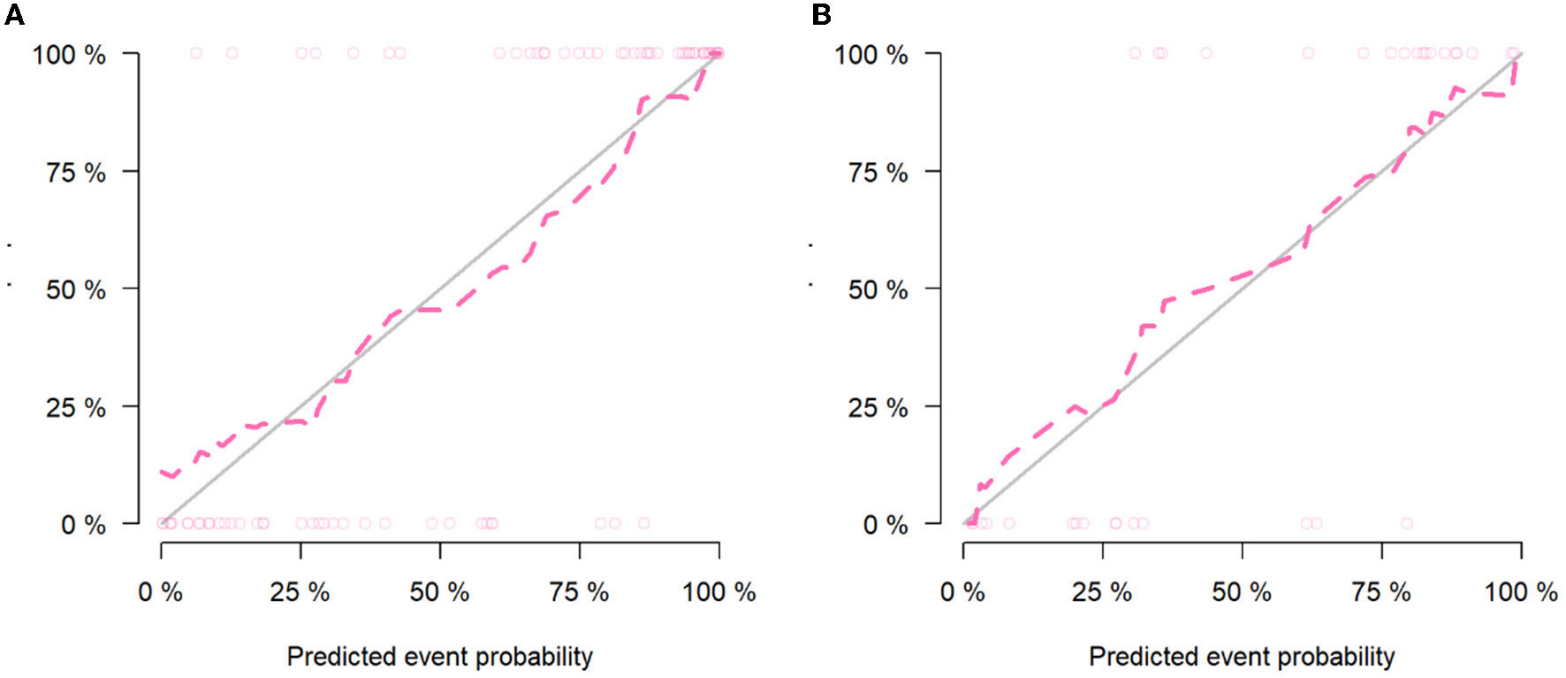
Calibration curves of the clinical-radiomics model in the training group **(A)** and validation group **(B)**.

**Figure 6:**
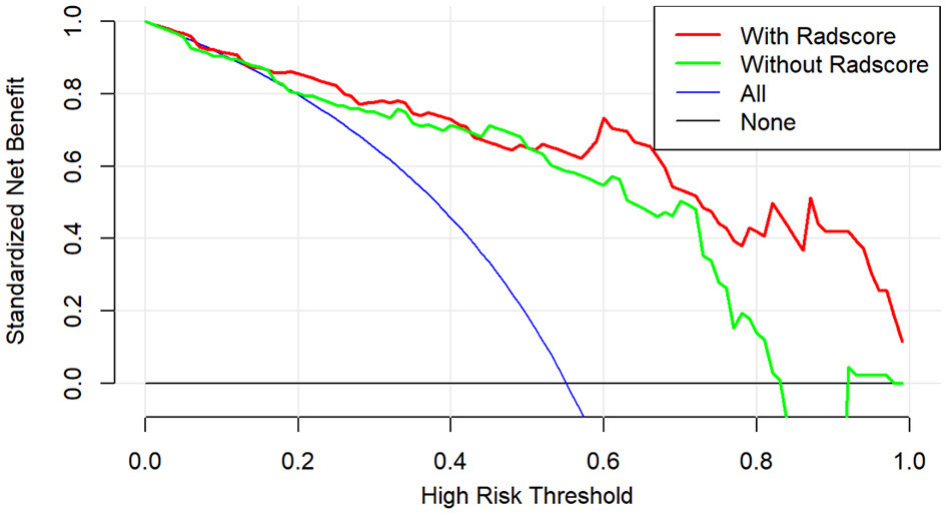
Decision curve analysis of clinical benefit for clinical application for the clinical-radiomics model.

The calibration curve results showed good consistency between the nomogram-predicted probability of ABE and the actual ABE observed in the training group and validation group (Hosmer–Lemeshow test; *P* = 0.137 and 0.362, respectively) (Figures 5A, B). The decision curve analysis applied to predict ABE is shown in Figure 6.

### Radiologists' visual diagnosis

Radiologists' information is shown in Table E2. The AUC of the visual diagnoses of radiologists one and two based on T1WI were 0.57 (*P* = 0.215, 95% CI: 0.46–0.68) and 0.63 (*P* = 0.019, 95% CI: 0.53–0.73), with a sensitivity of 0.377 and 0.459, specificity of 0.760 and 0.800, a positive predictive value of 0.657 and 0.737, and negative predictive value of 0.500 and 0.548, respectively. The AUC of the radiologists' final visual diagnosis was 0.66 (*P* = 0.003, 95% CI: 0.56–0.77), with a sensitivity of 0.508, specificity of 0.820, a positive predictive value of 0.775, and negative predictive value of 0.577. The ROC curves of radiologists' visual diagnosis are shown in Figure E2.

DeLong's test showed that the discriminative performance of the clinical model and clinical-radiomics model in the training and validation groups was increased compared to radiologists' visual diagnosis (Table 4).

**Table 4:** Comparison of discriminative performance between radiologists' visual diagnosis and models.

**Radiologist**	**Group**	**Model**	***P*-value**
Radiologist 1	Training group	Clinical model	<0.001^*^
		Rad_score	<0.001^*^
		Clinical-radiomics model	<0.001^*^
	Validation group	Clinical model	0.001^*^
		Rad_score	0.018^*^
		Clinical-radiomics model	<0.001^*^
Radiologist 2	Training group	Clinical model	0.003^*^
		Rad_score	0.002^*^
		Clinical-radiomics model	<0.001^*^
	Validation group	Clinical model	0.009^*^
		Rad_score	0.083
		Clinical-radiomics model	<0.001^*^
Radiologists' final visual diagnosis	Training group	Clinical model	0.013^*^
		Rad_score	0.009^*^
		Clinical-radiomics model	<0.001^*^
	Validation group	Clinical model	0.023^*^
		Rad_score	0.171
		Clinical-radiomics model	<0.001^*^

^*^p < 0.05.

## Discussion

Conventional MRI lacks quantitative indicators for brain damage in ABE, and it is impossible to objectively and comprehensively assess the risk of ABE, especially for neonates who may have brain damage when their GPs do not show hyperintensity on T1WI. In this study, we developed and validated a clinical-radiomics model based on T1WI for individualized prediction of ABE, which demonstrated good discrimination, calibration, and clinical benefit. It is worth noting that the discriminative performance of the clinical model and clinical-radiomics model in the training and validation groups was increased compared to that of radiologists with rich experience in pediatric radiology diagnosis. These results indicated that it is difficult to identify the ABE early based on T1WI by the naked eye. Moreover, the sensitivity of radiologists' visual diagnosis was low; however, the models were effectively improved. The nomogram was easy to use and may facilitate personalized risk stratification and further treatment decision-making for neonates with ABE, which could potentially provide a visualized and precise clinical support tool.

ABE is brain damage caused by unconjugated bilirubin passing through the blood-brain barrier (14). The neurotoxicity of unconjugated bilirubin is highly selective, implicating GPs, the substantia nigra, reticulata, subthalamic nuclei, brain stem, auditory, vestibular, and oculomotor nuclei, the hippocampus, and cerebellum (15); it particularly implicates GPs, which are related to its active metabolism (16). The mechanism of unconjugated bilirubin-induced neuron damage has not been fully elucidated. Existing hypotheses include excitotoxicity hypotheses (17), bilirubin-induced neuroinflammation (18, 19), and oxidative stress mechanisms (20). The destruction of bilirubin on neurons is recoverable in the early stages; consequently, early identification of ABE risk factors and intervention is an important method to prevent ABE, reduce the sequelae, and improve the prognosis.

Gestational week and the type of delivery were considered independent risk factors of ABE and they were incorporated into the clinical model. For neonates, the earlier the gestational week, especially gestational weeks earlier than 37 weeks, the higher the risk of ABE. This was due to the lower liver enzyme activity in premature infants, which affected the combination of human serum albumin and bilirubin, and premature infants were often treated with antibiotics, which destroyed the intestinal microecological environment. These factors caused bilirubin accumulation and led to ABE (21). The type of delivery also affected the risk of ABE; cesarean births were more likely to develop ABE, and this may be related to anesthesia (22, 23). In the radiomics model, the selected features were composed of the following categories: one first-order feature (Left_Skewness), two shape features (Right_SurfaceVolumeRatio and Left_Maximum2DDiameterRow), and seven texture features (Right_ RunEntropy, Left_Correlation, Right_SmallAreaLowGrayEmphasis, Right_Imc1, Right_Correlation, Left_GrayLevelNonUniformity_2, and Left_Idmn). Smaller skewness reflected the left deviation of the image, and more voxels were high intensity. Macroscopically, the higher intensity of left globus pallidus increased the risk of ABE. Smaller Left_Maximum2DDiameterRow and larger Right_SurfaceVolumeRatio were correlated closely with the risk of ABE. We speculated this was due to the neurotoxicity of unconjugated bilirubin increasing the influx of calcium ions, stimulating the activity of proteolytic enzymes, and leading to apoptosis (20, 24), changing the shape and volume of GP. The texture features described the distribution of voxel intensity and the spatial relationship between local adjacent voxels, which were a comprehensive reflection of the intrinsic properties of the image, and they could quantify the complexity of the texture of bilateral GPs in neonatal with ABE. However, associating a single texture feature with complex biological processes remains a challenge. These texture features included in the radiomics model may reflect the heterogeneity of bilateral GPs in neonatal with ABE. Liu et al. (25) also established models to distinguish between ABE and healthy neonates based on T1WI, and the best model obtained good diagnostic efficiency with an AUC of 0.946. Wu et al. (26) integrated multimodal MRI with deep-learning approaches to diagnose ABE, with the combination of three modalities, T1WI, T2WI, and diffusion-weighted imaging, with an AUC of 0.991 ± 0.007. However, these studies did not consider clinical factors. The most important thing is that they did not set up independent validation sets to verify the established models. Our study combined clinical and radiomics models and achieved a good prediction performance; the independent validation group verified the established model, and the risk of overfitting was effectively avoided.

Our study had several limitations. First, this was a retrospective study and potential bias may have been introduced in the selection of research subjects; some clinical data such as albumin and blood type were not considered. Furthermore, this batch of data was scanned using the same equipment, the sample size was small, there was a long inclusion period, and a lack of external validation; therefore, a larger cohort of prospective studies based on multicenter data is needed to verify the performance of the clinical-radiomics model. Lastly, the regions of interest in this study were manually placed, which may inevitably lead to certain errors. We have started to develop an automatic segmentation tool for GPs, which can segment the GPs more accurately and robustly in the future.

## Conclusions

In conclusion, we developed and validated a combined clinical-radiomics model based on T1WI to predict ABE. The application of the nomogram could potentially provide a visualized and precise clinical support tool.

## Data availability statement

The raw data supporting the conclusions of this article will be made available by the authors, without undue reservation.

## Ethics statement

The studies involving human participants were reviewed and approved by the First Affiliated Hospital of Dalian Medical University Ethics Committee. Written informed consent to participate in this study was provided by the participants' legal guardian/next of kin.

## Author contributions

JY: conceptualization, investigation, and data curation. YL: data curation and writing – original draft. YJ: validation and visualization. BG: data curation. JW: resources and supervision. YG: software and methodology. LX: methodology. YM: conceptualization, funding acquisition, and writing – review and editing. All authors contributed to the article and approved the submitted version.
