# Supplementary material for: Development and evaluation clinical-radiomics analysis based on T1-weighted imaging for diagnosing neonatal acute bilirubin encephalopathy
Source: Front Neurol. 2023 Feb 14;14:956975. doi: 10.3389/fneur.2023.956975 (PMC9971958; doi:10.3389/fneur.2023.956975)
Supplement: Supplementary file 1 [file Data_Sheet_1.docx]

**Supplemental Materials**

**Appendices E1**

**Table E1 Radiomics features**

| Shape  N=13 | Maximum 3D Diameter |
| --- | --- |
|  | Maximum 2D Diameter Slice |
|  | Sphericity |
|  | Minor Axis |
|  | Elongation |
|  | Surface Volume Ratio |
|  | Volume |
|  | Major Axis |
|  | Surface Area |
|  | Flatness |
|  | Least Axis |
|  | Maximum 2D Diameter Column |
|  | Maximum 2D Diameter Row |
| First Order  N=18 | Interquartile Range |
|  | Skewness |
|  | Uniformity |
|  | Median |
|  | Energy |
|  | Robust Mean Absolute Deviation |
|  | Mean Absolute Deviation |
|  | Total Energy |
|  | Maximum |
|  | Root Mean Squared |
|  | 90 Percentile |
|  | Minimum |
|  | Entropy |
|  | Range |
|  | Variance |
|  | 10 Percentile |
|  | Kurtosis |
|  | Mean |
| Gray-Level Co-occurrence Matrix (GLCM)  N=24 | Joint Average |
|  | Sum Average |
|  | Joint Entropy |
|  | Cluster Shade |
|  | Maximum Probability |
|  | Idmn |
|  | Joint Energy |
|  | Contrast |
|  | Difference Entropy |
|  | Inverse Variance |
|  | Difference Variance |
|  | Idn |
|  | Idm |
|  | Correlation |
|  | Autocorrelation |
|  | Sum Entropy |
|  | MCC |
|  | Sum Squares |
|  | Cluster Prominence |
|  | Imc2 |
|  | Imc1 |
|  | Difference Average |
|  | Id |
|  | Cluster Tendency |
| [Gray Level Dependence Matrix](http://www.baidu.com/link?url=QjYOXFFA_QvJVTK2pUEgbJgaze4zSOUA4fLjdTOALgjlYXlplmIe_U4DNN7dM1sPXrn-UQXYkEej2mS_lDy_w42t3q-VbT5ygijkalc4HDOuWw3ZQHLIA_QihP77GEAd) (GLDM)  N=14 | Gray Level Variance |
|  | High Gray Level Emphasis |
|  | Dependence Entropy |
|  | Dependence Non Uniformity |
|  | Gray Level Non Uniformity |
|  | Small Dependence Emphasis |
|  | Small Dependence High Gray Level Emphasis |
|  | Dependence Non Uniformity Normalized |
|  | Large Dependence Emphasis |
|  | Large Dependence Low Gray Level Emphasis |
|  | Dependence Variance |
|  | Large Dependence High Gray Level Emphasis |
|  | Small Dependence Low Gray Level Emphasis |
|  | Low Gray Level Emphasis |
| Gray-Level Run-Length Matrix (GLRLM)  N=16 | Short Run Low Gray Level Emphasis |
|  | Gray Level Variance |
|  | Low Gray Level Run Emphasis |
|  | Gray Level Non Uniformity Normalized |
|  | Run Variance |
|  | Gray Level Non Uniformity |
|  | Long Run Emphasis |
|  | Short Run High Gray Level Emphasis |
|  | Run Length Non Uniformity |
|  | Short Run Emphasis |
|  | Long Run High Gray Level Emphasis |
|  | Run Percentage |
|  | Long Run Low Gray Level Emphasis |
|  | Run Entropy |
|  | High Gray Level Run Emphasis |
|  | Run Length Non Uniformity Normalized |
| Grey-Level Size-Zone Matrix (GLSZM)  N=16 | Gray Level Variance |
|  | Zone Variance |
|  | Gray Level Non Uniformity Normalized |
|  | Size Zone Non Uniformity Normalized |
|  | Size Zone Non Uniformity |
|  | Gray Level Non Uniformity |
|  | Large Area Emphasis |
|  | Small Area High Gray Level Emphasis |
|  | Zone Percentage |
|  | Large Area Low Gray Level Emphasis |
|  | Large Area High Gray Level Emphasis |
|  | High Gray Level Zone Emphasis |
|  | Small Area Emphasis |
|  | Low Gray Level Zone Emphasis |
|  | Zone Entropy |
|  | Small Area Low Gray Level Emphasis |
| Neighborhood Gray Tone Difference Matrix (NGTDM)  N=5 | Coarseness |
|  | Complexity |
|  | Strength |
|  | Contrast |
|  | Busyness |

**Table E2 Radiologist information**

|  | **Reads per year(cases)** | **Experience(years)** | **Reads on ABE(cases)** |
| --- | --- | --- | --- |
| **Radiologist 1** | 1500-2500 | 15 | 150-250 |
| **Radiologist 2** | 2500-3000 | 20 | 200-300 |
| **Radiologist 3** | 3000-3500 | 25 | 300-400 |

Note.—ABE = acute bilirubin encephalopathy.

**Table E3 Detailed description of texture features included in radiomics model**

| **Texture feature** | **Description** |
| --- | --- |
| Run Entropy | Describe the disorder of run length, indicates the non-uniformity of texture in the image. |
| Correlation | Describe the similarity of gray level, reflects the local gray level correlation in the image. |
| Small Area Low Gray Emphasis | Describe the distribution of small connected and low gray level areas, indicative of finer textures and dark image. |
| Informational Measure of Correlation-1(Imc1) | Describe the correlation between the probability distributions using mutual information. |
| Gray Level Non Uniformity | Describe the non-uniformity of gray level in the image. |
| Inverse Difference Moment Normalized (Idmn) | Describe the variability of gray level in the image. |

**Figure E1**

**
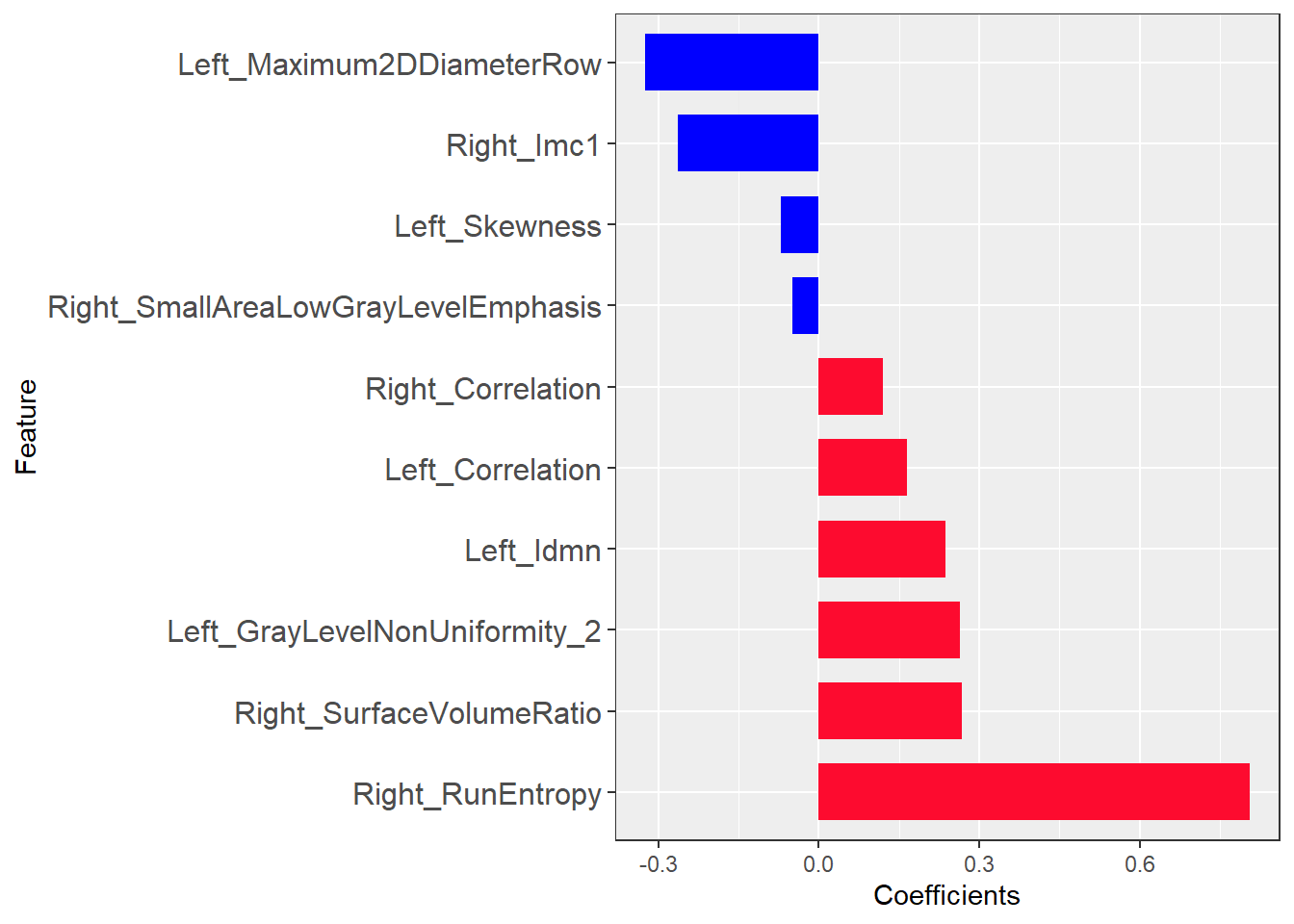
**

**Figure E1** The coefficients of the radiomics features in the multivariate logistic regression analysis of the radiomics model.

**Figure E2**

**
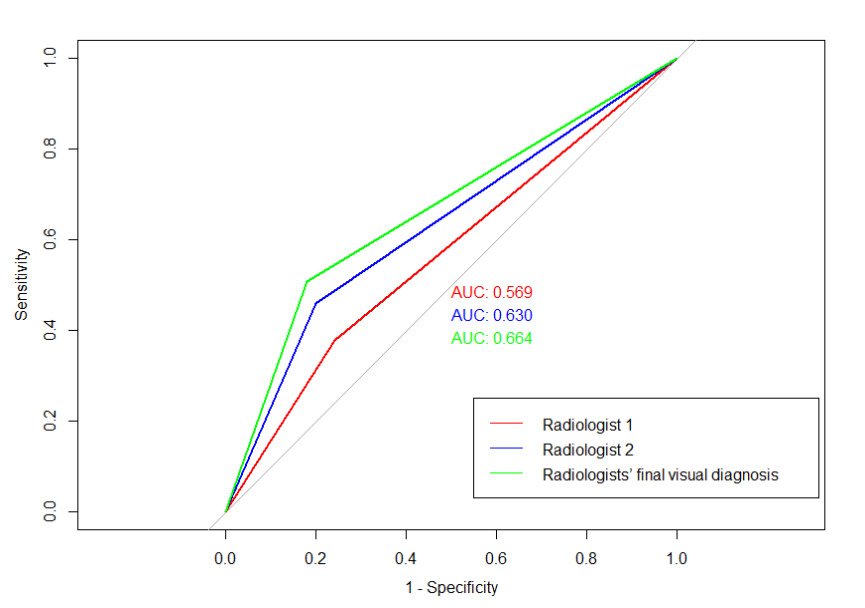
**

**Figure E2** ROC curves for the radiologist 1, radiologist 2 and radiologist’ final visual diagnosis.
